# MWCNTs Decorated with TiO_2_ as Highly Performing Filler in the Preparation of Nanocomposite Membranes for Scalable Photocatalytic Degradation of Bisphenol A in Water

**DOI:** 10.3390/nano13162325

**Published:** 2023-08-13

**Authors:** Antonio Tursi, Amerigo Beneduci, Isabella Nicotera, Cataldo Simari

**Affiliations:** 1 Department of Chemistry and Chemical Technologies, University of Calabria, Via P. Bucci, Cubo 15D, 87036 Arcavacata di Rende, Italy; antonio.tursi@unical.it (A.T.); amerigo.beneduci@unical.it (A.B.); isabella.nicotera@unical.it (I.N.); 2 SIRiA S.r.l.-Servizi Integrati e Ricerche per l’Ambiente, c/o Department of Chemistry and Chemical Technologies, Spin-Off of the University of Calabria, Via P. Bucci, Cubo 15D, 87036 Arcavacata di Rende, Italy; 3 National Reference Centre for Electrochemical Energy Storage (GISEL)—INSTM, Via G. Giusti 9, 50121 Firenze, Italy

**Keywords:** wastewater treatment, bisphenol A (BPA) removal, endocrine-disrupting chemicals, MWCNTs-TiO_2_ hybrid, nanocomposite membranes, visible-light photodegradation

## Abstract

Bisphenol A (BPA), an endocrine-disrupting compound with estrogenic behavior, is of great concern within the scientific community due to its high production levels and increasing concentration in various surface aquifers. While several materials exhibit excellent capacity for the photocatalytic degradation of BPA, their powdered nature and poor chemical stability render them unsuitable for practical application in large-scale water decontamination. In this study, a new class of nanocomposite membranes based on sulfonated polyethersulfone (sPES) and multiwalled carbon nanotubes decorated with TiO_2_ nanoparticles (MWCNTs-TiO_2_) were investigated as efficient and scalable photocatalysts for the photodegradation of BPA in aqueous solutions. The MWCNTs-TiO_2_ hybrid material was prepared through a facile and inexpensive hydrothermal method and extensively characterized by XRD, Raman, FTIR, BET, and TGA. Meanwhile, nanocomposite membranes at different filler loadings were prepared by a simple casting procedure. Swelling tests and PFG NMR analyses provided insights into the impact of filler introduction on membrane hydrophilicity and water molecular dynamics, whereas the effectiveness of the various photocatalysts in BPA removal was monitored using HPLC. Among the different MWCNTs-TiO_2_ content nanocomposites, the one at 10 wt% loading (sP-MT_10_) showed the best photoactivity. Under UV irradiation at 254 nm and 365 nm for 240 min, photocatalytic oxidation of 5 mg/L bisphenol A by sP-MT_10_ resulted in 91% and 82% degradation, respectively. Both the effect of BPA concentration and the membrane regenerability were evaluated, revealing that the sP-MT_10_ maintained its maximum BPA removal capability over more than 10 cycles. Our findings indicate that sP-MT nanocomposite membranes are versatile, scalable, efficient, and highly reusable photocatalysts for the degradation of BPA, as well as potentially for other endocrine disruptors.

## 1. Introduction

Nowadays, the global water system is highly compromised by poor sanitation, the absence of clean and safe resources, and the huge impact of chemicals, such as dyes, carcinogenic hydrocarbons, heavy metals, organic compounds, and pesticides, which undermine human health and the whole environment [1]. Most of these compounds generate serious repercussions on the systems of living beings; in particular, the endocrine-disrupting compounds (EDCs) alter the endocrine system, thereby creating havoc on biological systems [2]. These compounds have been constantly released into aquatic environments through different pathways: wastewater discharges, environmental leaching, diffusion through atmospheric phenomena, etc. [3]. To face the issues above, numerous water remediation technologies have been developed. For instance, natural materials and metal organic frameworks (MOFs) are widely acknowledged within the scientific community for their substantial potential across various applications. Notably, the utilization of natural substrates, including biochar [4,5], cellulose [6], activated carbon [7], and hyper-cross-linked polymers (HCP) [8], among others, demonstrates a robust adsorption capacity for diverse pollutant classes, such as fluorides [9,10], organic compounds, persistent organic pollutants (POPs) [11], dyes [12,13], and metals [14,15,16]. 

Among the EDCs, bisphenol A (BPA) stands out as a highly representative compound. Notably, BPA exhibits estrogenic characteristics and has garnered significant attention due to its high production, wide applications, and consistently high concentrations in various surface aquifers [17]. Bisphenol A, also known as 2,2-bis(4-hydroxyphenyl)propane, is a synthetic phenolic compound commonly utilized as an additive, flame retardant, or starting monomer for the production of epoxy resins and polycarbonate. Since the 1940s, BPA has found extensive use in the industrial production of plastic containers, DVDs, can linings, and thermal papers. The global production of BPA reaches approximately 8 million tons, solidifying its status as one of the most highly produced chemicals worldwide [18,19]. Additionally, the degradation processes of polyurethane plastics can lead to the release of BPA into the environment [20,21].

A major source of BPA is identified as flows from wastewater treatment plants to groundwater, leading to its spread to secondary sources, such as groundwater, surface water, and soil. Other sources are identified from untreated sewage and sludge use from WWTP in cropland, etc. [22,23]. Moreover, the escalating volumes of industrial and household waste deposited in landfills contribute to a mounting generation of landfill leachates containing elevated BPA concentrations (reaching up to 4500 µg/L), which subsequently infiltrate surface water and groundwater. Overall, BPA concentrations in groundwater range from 0.09 × 10^−3^ to about 230 µg/L [22]. For example, high concentrations of BPA have also been found in waste paper recycling plants (up to 370 μg/L) [24] and in bottled water in France (0.07–4.21 μg/L) [25]. In many cases, the maximum allowable concentrations of BPA for drinking water, set by government organizations and countries, have even been exceeded. Unfortunately, a critical issue that is difficult to solve immediately is due to the fact that many underdeveloped regions, such as Latin America and Africa, currently do not yet have a regulation for these EDCs in the aquifer sector [22]. All in all, a huge risk of long-term exposure of humans to BPA exists mostly due to the fact that current treatment technologies fail in completely removing this compound from drinking water [26]. Consequently, a relatively vast literature exists about the correlation between BPA exposure and effects on health: metabolic disorders, such as neurotoxicity, obesity, infertility, and precocious puberty, immune system alterations, and interference in cell development are all related to BPA absorption into the body. Furthermore, it has been demonstrated that cardiovascular disease, diabetes in adults, and endometrial hyperplasia are other disorders associated with BPA exposure [27]. In view of such serious problems that the diffusion and adsorption of BPA causes to humans, several technologies have been studied for BPA removal, including coagulation/flocculation/sedimentation/filtration [28,29,30], carbon nanomaterials (CNMs) (e.g., carbon nanotubes (CNTs)) [31,32] and graphene oxides (GOs) [33,34]), membrane filtration [35,36], ultraviolet (UV) irradiation [37,38], metal-free modification of porphyrin-based porous organic polymers for effective photocatalytic degradation of bisphenol A in water [34], biological processes [39,40], and advanced oxidation techniques [41,42,43]. However, despite their high sustainability, these materials may experience degradation or a decline in adsorption performance with repeated use cycles. Consequently, the development of an effective and sustainable method to remove BPA from contaminated waters is still a significant challenge for the scientific community.

Against this background, UV degradation is generally considered an effective method for the removal of these classes of contaminants from aqueous media. Moreover, the low energy consumption, correlated to a high degradation capacity without the use of chemical substances harmful to the environment and human health, hopefully allows for the potential application of this technology on a large scale [44,45,46,47]. To date, a large number of emerging photocatalysts have been explored, including Bi2WO6, Co-doped BiOCl, and Ag3PO4/rGH, demonstrating excellent photocatalytic activity during the degradation of BPA [48,49,50,51]. However, the harsh and costly conditions for their synthesis, combined with the poor chemical stability, limit the usefulness of these catalysts in practical applications. Consequently, titanium dioxide (TiO_2_) is still regarded as the dominant photocatalyst for environmental applications due to its superior photocatalytic oxidation behavior, non-toxicity, low environmental impact, photostability, and inexpensiveness [52]. Unfortunately, the photocatalytic performance of TiO_2_ in the bulk form generally suffers from serious catalyst agglomeration, insufficient visible-light absorption, and rapid charge recombination [44,46,53]. For instance, TiO_2_ in the anatase form (that is, the phase for TiO_2_ exhibiting the highest photocatalytic activity) has a band gap Eg of about 3.2 eV [54]. This means that e/h^+^ pairs could be produced only through the absorption of light with a wavelength below 380 nm [55]. In attempting to enhance its light-harvesting efficiency while simultaneously improving the photocatalytic performance, a large number of different strategies have been explored by worldwide researchers, including chemical doping, dye sensitization, and heterojunction construction [44,46,52,53,56]. In the past few years, the design and development of a TiO_2_-hybrid composite photocatalyst have attracted a great amount of research interest. In this regard, due to their prominent structural and electric properties, carbon-based materials, such as graphene, carbon nanotubes, and fullerenes, have demonstrated tremendous potential in extending the photo-response range of TiO_2_ to the visible-light region through the modification of the bandgap and/or sensitization [57]. Among them, carbon nanotubes (CNTs) hold great promise due to their capability to conjugate a very large specific surface area with excellent electronic mobility [58]. Indeed, intertwined carbon nanotubes easily generate a highly conductive network with fast interfacial polarization [59], which makes them highly attractive in the enhancement of the visible-light-driven photoactivity of TiO_2_. Ou and co-workers successfully demonstrated that multiwalled carbon nanotubes (MWCNTs) are able to act as photosensitizers in the MWCNTs-TiO_2_:Ni composite catalyst. The latter was then successfully used for photocatalytic H2 generation under visible light illumination [60]. Dai et al. synthesized a MWCNT-TiO_2_ nanohybrid catalyst with visible-light-driven photoactivity through the direct growth of TiO_2_ nanoparticles on the surface of the MWCNTs. The hybrid material exhibited high photocatalytic activity under visible light irradiation during the hydrogen generation process [61]. Despite such a huge potential, however, to date and to the best of our knowledge, the use of a MWCNTs-TiO_2_ catalyst for the photocatalytic degradation of BPA has been completely inedited. Additionally, during the evaluation of new photocatalytic materials for practical applications, one of the major issues is related to the difficulty in recovering and regenerating the photocatalyst after the photocatalytic process [62,63,64]. As such, any new materials should be able to provide both good photocatalytic performance and recyclability. 

To meet these challenging requirements, we propose the preparation of a composite membrane incorporating a novel MWCNTs-TiO_2_ nanohybrid catalyst. This strategy should allow for the combination of the typical advantages of membrane technology, i.e., flexibility, scalability, modular design, and ease in preparation and maintenance, with the photocatalysis, thus providing the most appropriate solution to the industrial requirements for large-scale application. It is noteworthy that the composite membrane can be easily removed from water and regenerated for reuse after the photocatalytic process. To date, only very few studies have reported the use of nanocomposite membranes with photocatalytic activity obtained through the incorporation of TiO_2_ [65,66] or TiO_2_-based nanostructures [67], and the approach has been demonstrated to be very promising. Furthermore, it was recently demonstrated that the degradation of water pollutants, specifically dye molecules, can be further enhanced by using sulfonated polymers as the hosting matrix due to a synergistic effect of adsorption and photocatalytic reaction [68,69]. Based on the above, in this study, the sulfonated derivative of polyethersulfone (sPES) was selected as the hosting matrix on account of its low cost, large commercial availability, low environmental impact, excellent film-forming ability, and superior chemical and thermo-mechanical resistance [70]. Chlorosulfonic acid was used as the sulfonating agent because it allowed for fast and relatively easy reaction control. Also, this synthetic approach is generally free from side reactions and cleavage of the polymer backbone [71]. The reaction was finely controlled in order to reach an ion exchange capacity (IEC) of circa 3 meq/g. At this IEC, the best compromise between good hydrophilicity and mechanical robustness is generally achieved [70]. Therefore, in this work, the MWCNTs-TiO_2_ hybrid material was first prepared through the most popular hydrothermal method by using titanium(IV) butoxide as a relatively inexpensive titanium source, and it was tested for its potential in BPA removal from aqueous solutions. To clarify the microstructural and chemical properties of the hybrid material, SEM, XRD, Raman, FTIR, TGA, and porosimetry analyses were carried out, demonstrating that the surface of the MWCNTs was successfully decorated with TiO_2_ nanoparticles. Thereafter, sPSU/MWCNTs-TiO_2_ nanocomposite membranes at various filler loadings were prepared by simple solution intercalation. The mechanical performance of the nanocomposite membranes was investigated by dynamic mechanical analysis, while their hydrophilic behavior was characterized by swelling tests and the Pulse Field Gradient NMR method. The latter technique allowed for direct measurement of the self-diffusion coefficients, thus providing crucial information on the effect of the filler introduction on the microstructure of the water-filled cluster of the resulting nanocomposites. The photocatalytic degradation of BPA was carried out by batch tests using different experimental conditions (variation of wavelength of UV rays, catalyst dose, initial concentration of BPA, etc.). Additionally, the possibility of reusing the nanocomposite membranes after numerous cycles of degradation was also evaluated. The preliminary results demonstrated that the preparation of sPSU/MWCNTs-TiO_2_ nanocomposite membranes could be exploited as a simple, scalable, effective, low-cost, and low-impact method to remove BPA from primary and secondary sources of contamination in various surface water compartments. As such, this study is of great importance for the protection of health in both humans and the environment.

## 2. Materials and Methods

### 2.1. Materials

The following reactants were all supplied by Sigma-Aldrich (Sigma-Aldrich, Milan, Italy) and used without any further purification/modification: Multi-walled carbon nanotubes (MWCNTs, powder with particle size <20 mm), titanium(IV) butoxide (reagent grade, 97%), N,N-dimethylacetamide (anhydrous, 99.8%), sulfuric acid (95–97 wt%), isopropyl alcohol (ACS reagent, ≥99.5%), and nitric acid (65 wt%). 

The sPES was synthesized according to the procedure reported elsewhere [70], and the experimental conditions were finely adjusted to achieve an ion exchange capacity of 3.19 meq/g.

### 2.2. Oxidation of Multi-Walled Carbon Nanotubes (oxMWCNTs)

Pristine MWCNTs were suspended in a H_2_SO_4_/HNO_3_ mixture (3:1 *v*/*v*) and subjected to sonication for 3 h at RT. Finally, the dispersion was centrifuged, washed several times with deionized water, and dried in an oven at 60 °C overnight to recover the oxidized MWCNTs (oxMWCNTs) in a fine powder. 

### 2.3. Surface Decoration of MWCNTs with Titania Nanoparticles (MWCNTs-TiO_2_)

**Scheme 1:**
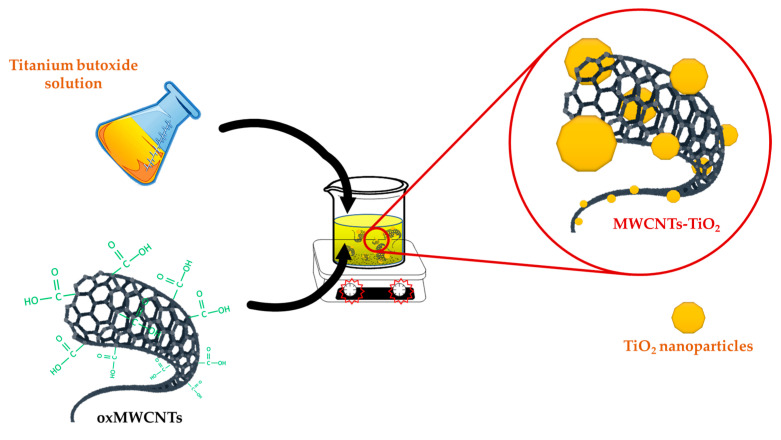
Experimental procedure for the synthesis of the MWCNTs-TiO_2_ nanohybrid material.

A one-pot hydrothermal method was exploited to synthesize a MWCNTs-TiO_2_ hybrid material. Titanium(IV) butoxide was used as a Titania precursor [72], and the synthesis pathway is illustrated in Scheme 1. In a beaker, titanium(IV) butoxide was mixed with nitric acid, isopropyl alcohol, and water, and the reaction was heated at 60 °C using an oil bath. After 1 h of stirring, a titanium(IV) ionic (Ti^4+^) solution was formed. Subsequently, a homogenous aqueous dispersion of oxidized MWCNTs was gradually added, and the mixture was stirred at 60 °C for an additional 18 h. The molar ratio between the titanium butoxide and GO was 14:1 (*w*/*w*). The reaction was quenched by pouring it into ice-cold ethanol. Following centrifugation (15,000 r/min), the MWCNTs-TiO_2_ hybrid material was recovered as a fine black powder, which underwent several washes with deionized water before being vacuum dried at 60 °C overnight. 

### 2.4. Preparation of the Nanocomposite Membranes

Nanocomposite membranes were prepared using a straightforward solution-casting procedure [73]. A pristine membrane was prepared by dissolving sPES powder in N,N-dimethylacetamide (15 wt% solution). Once a clear solution was achieved, it was cast onto a Petri dish and introduced into an oven set at 60 °C, allowing complete solvent evaporation. Nanocomposite membranes were prepared by directly incorporating the MWCNTs-TiO_2_ nanohybrid into the sPES-DMAc polymer solution. To obtain a macroscopically homogeneous dispersion, vigorous mechanical stirring and sonication were alternated for 2 days at room temperature. The loading of the filler ranged from 1 to 10% by weight with respect to the polymer. It should be noted that membranes with higher filler contents exhibited inhomogeneity and brittleness due to particle agglomeration. The final dispersion was then cast on a Petri dish at 60 °C until complete evaporation of the solvents. The resultant membranes were designated as sP-MT_1_, sP-MT_5_, and sP-MT_10_ according to the filler amount, i.e., 1, 5, and 10 wt%, respectively. Before the characterizations, all membranes underwent chemical activation in a 1 M H_2_SO_4_ solution at 60 °C for 15 h followed by thorough rinsing in deionized water to eliminate any residual traces of acid. All the sPES-based membranes had a dry thickness of 55 ± 5 μm.

### 2.5. Characterization

X-ray diffraction (XRD) measurements were carried out on a Bruker AXS Diffractometer/Reflectometer (D8) furnished with a Dynamic Scintillation Detector, NaI, and a Gobel mirror. The XRD spectra were acquired using the Cu-Kα radiation in transmission mode on powder samples. Prior to analysis, the powders were meticulously inserted into glass capillaries (ϕ = 1 mm, Hilgenberg GmbH, Strauchgraben, Malsfeld, Deutschland), which were then positioned in a sample holder [74,75]. Spectra were collected at room temperature within the 2*θ* range of 5° to 40°, with increments of 0.03° and a counting time of 1 s/step. 

The FTIR spectra were recorded using a Perkin-Elmer Spectrum GX instrument (Perkin-Elmer, Waltham, MA, USA) with KBr pellets. The acquisition encompassed the range of 400–3600 cm^−1^ at 2 cm^−1^ resolution, achieved by averaging 64 scans. This allowed for the improvement of the signal-to-noise ratio [76]. 

Raman spectra were acquired on a Jobin Yvon micro-Raman LABRAM apparatus (Horiba Ltd., Kyoto, Japan) using the 632.8 nm wavelength of a He–Ne laser as the excitation source. Equipped with a 50× Olympus lens objective (focal length of 15 mm), the instrument power was about 5 mW and focused on a spot of about 5 μm in diameter. The Raman spectra were gathered within the 200 and 3900 cm^−1^ range. Changes in the Raman spectra were used to explore molecular-level interactions within the MWCNTs-TiO_2_ derivatives [77]. 

A TG-7 instrument from Perkin-Elmer was employed for thermogravimetric analyses (TGA). Samples were first dried at 60 °C for 24 h and then positioned in an alumina crucible. The heating process took place over a temperature range of 50–900 °C at a rate of 10 °C/min, utilizing flowing nitrogen [78]. 

N_2_ adsorption/desorption isotherms were acquired at 77 K via a Micromeritics ASAP 2460 apparatus (Micromeritics Instrument Corporation, Norcross, GA, USA ). Before each adsorption measurement, the sample underwent outgassing at 473 K for 12 h. Density Functional Theory (DFT) was used to calculate the pore size distributions (PSD) [79].

A Bruker AVANCE 300 wide-bore spectrometer (Bruker Corporation, Billerica, MA, USA) working at 300 MHz on ^1^H was used for the NMR measurements. The instrument was equipped with a Diff30 Z-diffusion 30 G/cm/A multinuclear probe with substitutable RF inserts. In particular, a pulsed field gradient stimulated-echo (PFG-STE) sequence [80] was used to obtain a direct measurement of the self-diffusion coefficients (*D*) of water confined within the hydrophilic clusters of the various membranes. For this set of experiments, the Pulse gradient length (*δ*) was 0.8 ms, while the diffusion delay time (Δ) was 8 ms. The gradient amplitude (*g*) ranged from 80 to 800 G/cm. The uncertainties in D values were calculated to be circa 3% on the basis of the very low standard deviation of the fitting curve and the repeatability of the measurements. Diffusivity measurements were collected under increasing temperature in the range of 20–130 °C each 20 °C, with 15 min as the equilibration time for each temperature. Details on the NMR sample preparation can be found elsewhere [81].

The thermomechanical performance of the membranes was investigated using dynamic mechanical analysis (DMA) on a Metravib DMA/25 analyzer (Metravib Limonest, France) equipped with a shear jaw for clamping the films [82,83,84]. Rectangularly shaped samples were directly cut from the membranes and subjected to a dynamic stress of amplitude 10^−4^ at 1 Hz. The temperature ranged from 25 to 180 °C, with a heating rate of 2 °C/min.

### 2.6. BPA Analytical Determination

The bisphenol A concentration in the filtrate samples was measured by means of a high-performance liquid chromatography (HPLC) system coupled with a diode array detector (SPD-20A Prominence UV Detector, SIL-20AC Prominence autosampler, CTO-20AC Prominence Column oven, DGU-20A5 Prominence degasser, LC-20AT pump, Shimadzu, Tokyo, Japan). The samples were separated by reverse phase (RP) chromatography using a C18 Luna (Phenomenex, Torrance, CA, USA) column (5 µm, 250 × 4.6 mm). The HPLC method was obtained by Tursi et al. [85]. The mobile phase was a mixture of water/acetonitrile (35/65, *v*/*v*) at a flow rate of 1 mL/min using a UV wavelength at 227 nm. The solvents were eluted isocratically, and the injection volume was kept at 20 µL. A calibration curve was prepared for the quantification of BPA in water by using standards at different concentrations (from 0.5 ppm to 50 ppm). No traces of byproducts amenable to polymer degradation and/or the release of impurities from the MWCNTs-TiO_2_ nanomaterial were detected by HPLC analysis. 

### 2.7. Preliminary Photocatalytic Degradation Experiments of BPA in Aqueous Batches 

The photocatalytic activity of the MWCNTs-TiO_2_ membranes was studied by carrying out several batch experimental runs. The experimental temperature was 20 °C. The surface of the MWCNTs-TiO_2_ membranes was approximately 2.5 cm^2^. In order to evaluate the photocatalytic performances of MWCNTs-TiO_2_ membranes, preliminary tests were conducted under the irradiation of a suitable UV lamp (6 W, 254 nm–365 nm) at a distance of 5 cm from the aqueous batch polluted with different initial concentrations of BPA ranging from 5 to 50 ppm (V = 50 mL, pH = 5.5). During the UV light irradiation experiments, constant stirring at 100 rpm was maintained by an orbital stirring plate (Orbital Platform Shaker, PSU-10i, Grant Instruments, Cambridge, UK) to ensure the tested solution was homogeneous. 

A preliminary investigation of BPA degradation was carried out using different experimental conditions in order to evaluate the degradation efficiency of UV rays at 254 and 365 nm. Further tests were conducted in order to evaluate the effect of the dose (%) of TiO_2_ catalyst on the membranes and the effect of the initial concentration of BPA on its degradation under UV light. All batch tests were performed under the same conditions of volume (V = 50 mL) and pH (5.5).

During all tests, a fixed amount of sample was withdrawn from polluted batches at different time intervals over 240 min and analyzed according to the method reported in the previous paragraph. All experiments were repeated three times to observe the reproducibility of the measurements, allowing for the attribution of a standard deviation. The removal efficiency (RE%) of the treatments was calculated using the following equation (Equation (1)):

(1) $$RE\%=Percentage\;photodegradation\;of\;BPA=\frac{C_{0\;}-C}{C_{0}}\times 100$$

where “*C*_0_” is the initial concentration of BPA and “*C*” is the concentration of BPA at time ‘t’.

## 3. Results and Discussion

### 3.1. Characterization of the MWCNTs-TiO_2_ Nanohybrid

**Figure 1:**
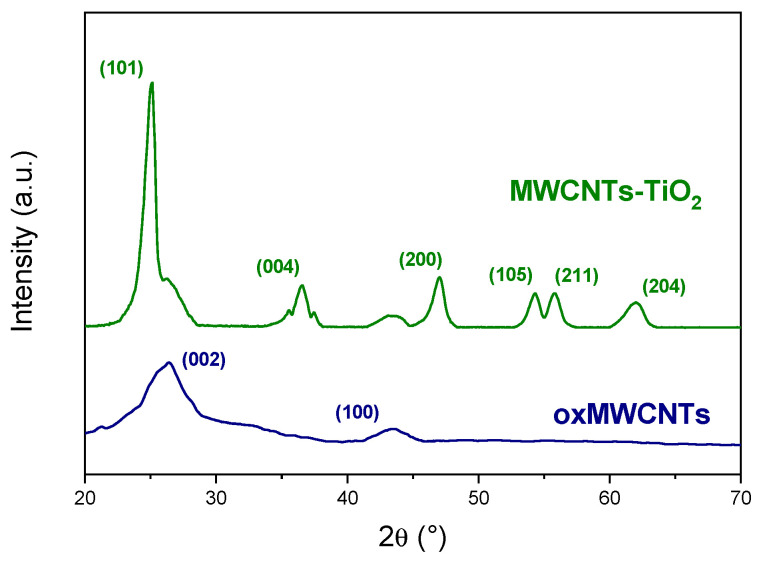
XRD patterns of oxMWCNT and MWCNT-TiO_2_ materials.

The XRD patterns of oxidized MWCNTs and MWCNTs-TiO_2_ materials are depicted in Figure 1. The oxMWCNTs exhibit distinctive diffraction peaks at 2*θ* = 26.2° (002) and 2*θ* = 43.5° (100), which are attributed to the hexagonal honeycomb structure of carbon nanotubes [86]. In contrast, the X-ray diffraction pattern of the hybrid material displays several additional peaks, indicating the successful growth of TiO_2_ onto the CNTs’ surface. Notably, a sharp peak at 2*θ* = 25.1° (101) indicates the presence of a crystalline anatase phase in the synthesized TiO_2_ particles. Peaks at (004), (200), (105), (211), and (204) further confirm that all of the crystal phases are anatase, because no peak of rutile appears [72]. The broadening of the (101) anatase peak was employed to calculate the average crystal size using the Scherrer equation [87], giving an average grain size of ca. 80 nm for TiO_2_. Likely, the presence of MWCNTs hinders direct grain-to-grain contact, resulting in the formation of such a small crystal size of Titania particles in the hybrid MWCNts-TiO_2_ material. 

**Figure 2:**
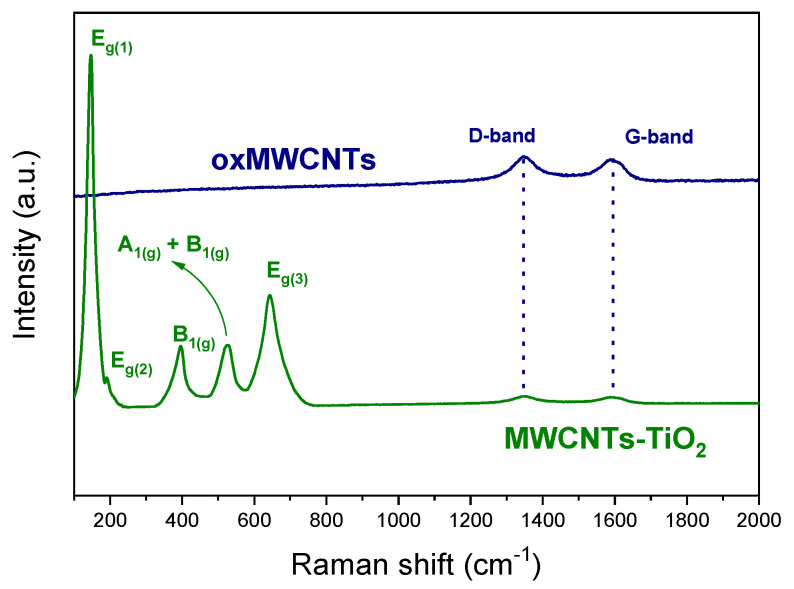
Raman spectra of oxMWCNT and its functionalized derivative.

The Raman analysis (Figure 2) further validates the successful formation of the MWCNTs-TiO_2_ nanohybrid. Indeed, the Raman pattern of MWCNTs-TiO_2_ exhibits characteristic bands of carbon nanotubes (namely, the D-band and G-band at 1345 and 1596 cm^−1^ [88], respectively), along with signals corresponding to anatase TiO_2_. These anatase TiO_2_ signals encompass E_g(1)_, E_g(2)_, B_1g_, A_1g_ + B_1g_, and E_g(2)_ modes [89]. It is worth noting that the latter signals display a slight shift compared to the typical bands of pure TiO_2_, confirming the chemical nature of the interaction between the MWCNTs and the TiO_2_ heterojunction composite [90]. 

**Figure 3:**
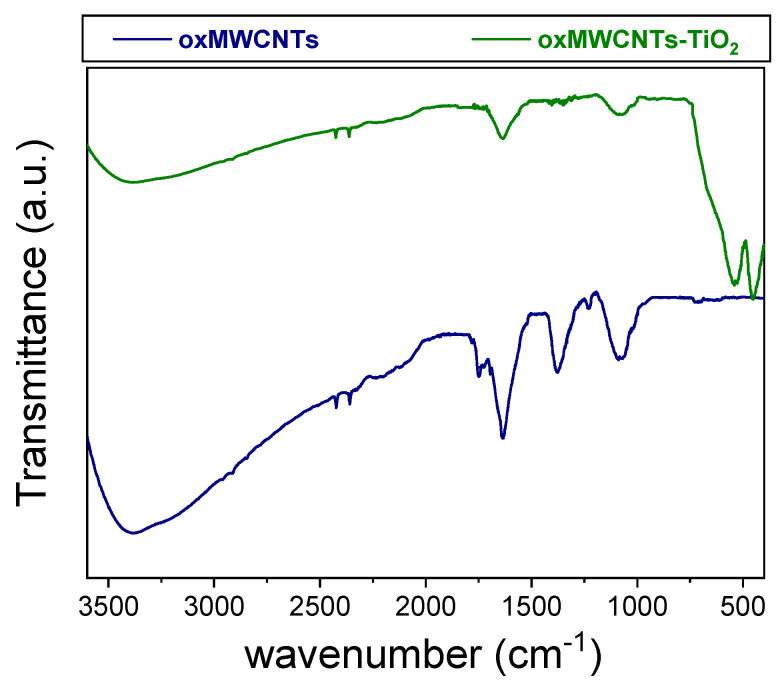
FTIR spectra of oxMWCNTs and MWCNTs-TiO_2_ samples.

The FTIR analysis was carried out to definitively confirm the nature of the interaction of MWCNTs with TiO_2_ nanoparticles. Figure 3 juxtaposes the FTIR spectrum of the hybrid material with that of parental oxMWCNTs in the wavenumber range of 400–3750 cm^−1^. The spectrum of MWCNTs reveals clear evidence of oxygen-containing functional groups resulting from oxidation in sulfuric/nitric acid [91]. Indeed, signals at 1070 and 1240 cm^−1^ correspond to epoxy (C-O, C-O-C) groups, while the adsorption bands at 1384, 1745, and 3380 cm^−1^ prove the presence of hydroxyl (C-OH) and carboxylic (-C-OOH) groups on the surface of MWCNTs. As can be seen, following the immobilization of TiO_2_ nanoparticles on the MWCNT sidewalls, the intensity of the characteristic resonance for carboxylic functionalities decreases, suggesting their consumption during the reaction with the Titania precursor. This undeniably proves the covalent linkage between oxidized MWCNTs and Titania, which likely results from the esterification between the COOH of oxidized MWCNTs and the -OH of TiO_2_ [92]. In addition, two absorbance resonances at 460 cm^−1^ and 530 cm^−1^ appear in the spectrum of MWCNTs-TiO_2_, which are credited to Ti–O–C stretching and the Ti–O-Ti vibration modes, respectively [93].

**Figure 4:**
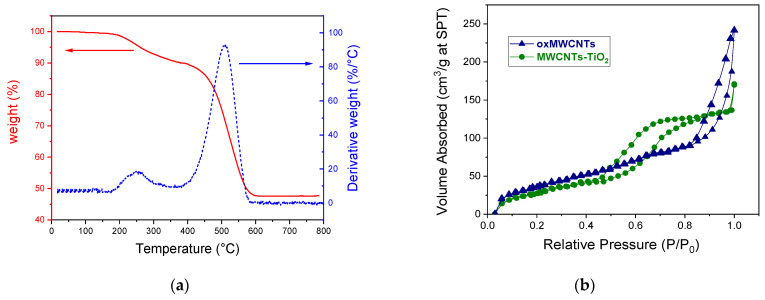
(**a**) TGA-DTG curves representing the thermal decomposition of the MWCNTs-TiO_2_ hybrid; (**b**) N_2_ adsorption–desorption isotherms of oxMWCNTs and MWCNTs-TiO_2_ samples.

The thermogravimetric analysis of the TiO_2_ derivative of the MWCNTs enabled the estimation of the TiO_2_ content in the nanohybrid materials. Figure 4a illustrates TGA and DTG curves in a temperature range of 20–800 °C. The first degradation step for MWCNTs-TiO_2_ material starts at ca. 200 °C due to the decomposition of oxygen-containing groups, while the critical temperature for the deterioration of the honeycomb structure is around 500–600 °C. Beyond this temperature range, the residual weight, indicative of TiO_2_ content, is 47.6%. 

Figure 4b presents the N_2_ adsorption–desorption isotherm of the MWCNTs-TiO_2_ material in comparison with oxidized MWCNTs. Both samples exhibit typical type IV isotherms, with narrow necks and wider bodies. Notably, oxMWCNTs present a hysteresis loop at a relative pressure (P/P_0_) ranging between 0.8 and 1.0, whereas it is shifted at a lower relative pressure in the case of MWCNTs-TiO_2_, i.e., 0.45–0.85. This suggests a huge difference in the mesoporous structure of the two materials. Oxidized MWCNTs exhibit a BET surface area of 187 m^2^ g^−1^, but it increases until 205 m^2^ g^−1^ in the hybrid MWCNTs-TiO_2_ material due to the anchoring of the TiO_2_ nanoparticles. This should enable higher photocatalytic activity. Conversely, the presence of TiO_2_ nanoparticles leads to a reduction in pore diameter, which is 43.11 nm for oxMWCNTs and 9.15 for MWCNTs-TiO_2_.

### 3.2. Nanocomposite Membranes

Following the successful synthesis of the MWCNTs-TiO_2_ nanohybrid, its potential as an additive for the preparation of sPES-based nanocomposite membranes for water decontamination was explored. Membranes were prepared via simple solution intercalation, and filler content was varied from 1 to 10 wt% with respect to the polymer to elucidate the effect of the filler content. The SEM investigation revealed that the sPES has a compact and uniform cross section devoid of fractures or defects (see Figure S1). Importantly, the introduction of the MWCNTs-TiO_2_ hybrid material does not induce any alteration in the microstructure of the resulting membrane. Even at the highest filler content (i.e., 10 wt%), there is no evidence of particle aggregation, proving that the filler maintains sub-nanometric dimensions. This suggests that the coupling of MWCNTs and TiO_2_ prevents the aggregation of TiO_2_ particles, thereby preserving their capacity to adsorb/degrade organic pollutants.

**Figure 5:**
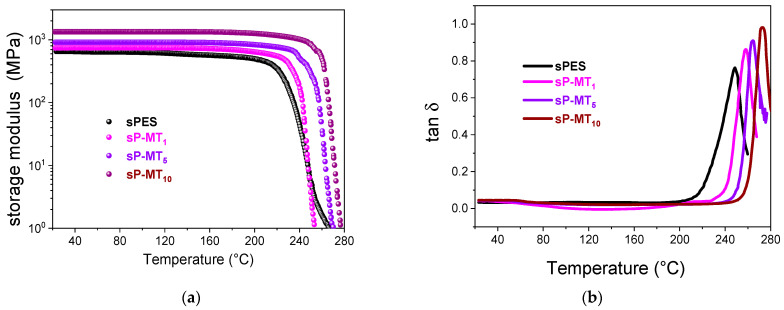
Temperature variation of (**a**) the storage modulus and (**b**) the tan δ for the sPES-based membranes.

For membrane applications in water treatment, adequate mechanical and thermal resistance is imperative. Accordingly, the viscoelastic properties of the sPES-based membranes were investigated by dynamic mechanical analysis (DMA). The temperature evolution of the storage modulus (E′) is illustrated in Figure 5a for membranes with varying filler loadings. It is well known that sPES possesses notable mechanical robustness [70], showcasing a substantial storage modulus of approximately 643 MPa. Furthermore, the introduction of the MWCNTs-TiO_2_ produces a significant enhancement in the mechanical strength of the nanocomposite membranes. The sP-MT_10_ membrane exhibits the highest E′ value, reaching 1345 MPa, which represents a two-fold increase compared to the pristine membrane. It is worth noting that the addition of the nanohybrid material also imparts positive effects on the thermal resistance of the resulting films. The storage modulus remains stable until 200 °C for all the membranes, and then it starts to decrease due to softening of the polymer chains. However, this critical temperature is progressively shifted toward a higher value as the filler content increases. The temperature variation of the tan δ (Figure 5b) better elucidates the relationship between the thermal resistance and the filler content. The tan δ profile is characterized by a single peak in the high temperature region, which is typically attributed to the α transition (Tg) of hydrophilic clusters within the polymer membrane. The Tg for the pristine sPES is 240 °C, but it increases to 273 °C, as in the case of sP-MT_10_. This substantial enhancement in thermo-mechanical resistance is likely amenable to the peculiar architecture of the MWCNTs-TiO_2_ material, which combines the reinforcement features of both very long nanotubes and TiO_2_ nanoparticles. As a result, the sP-MT membranes demonstrate a commendable capability to withstand severe operating conditions.

**Figure 6:**
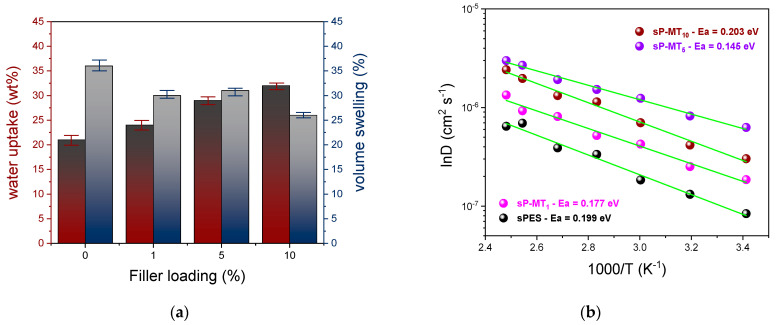
(**a**) Effect of filler content on water uptake and volume swelling of sPES-based nanocomposites; (**b**) Arrhenius plot of the self-diffusion coefficient values for the sPES and nanocomposite membranes. Green lines in the plot represent linear fitting to the experimental data.

The hydrophilic properties of the various sPES-based membranes were monitored by examining their water uptake and volume swelling, with the results illustrated in Figure 6a. Given the inherently hydrophilic nature of the MWCNTs-TiO_2_ material, water uptake increases with filler loading, culminating in a value of 32 wt% for the sP-MT_10_. Intriguingly, the volume swelling exhibits an inverse trend: higher filler content corresponds to reduced volume swelling. Likely, the long CNTs decorated with TiO_2_ nanoparticles enable the formation of a physically interconnected network at the microscale. This network minimizes dimensional fluctuations of the hydrophilic clusters in the nanocomposite membranes. Moreover, a deeper understanding of the structure–performance relationship for the sPES-based membranes could be gleaned from the investigation of the water dynamics through direct measurements of the self-diffusion coefficient [94,95]. Figure 6b illustrates the Arrhenius plot, which encompasses the temperature range of 20–130 °C and depicts the diffusion of water within the hydrophilic cluster of the various samples. The amount of water adsorbed is the main factor governing the self-diffusion coefficients [96]. As such, the nanocomposites exhibit higher diffusivity compared to the bare polymer. Additionally, D increases with the filler content until 5 wt%, and the fastest diffusivity of 6.29 × 10^−7^ cm^2^ s^−1^ is achieved at 20 °C by the sP-MT_5_ membrane. Higher loadings have detrimental effects on water mobility, which is likely due to the progressive obstruction of the hydrophilic channels in the nanocomposite membrane. Indeed, the activation energy (E_a_) associated with water mobility inside the membranes indicates that sP-MT_10_ exhibits the highest E_a_ value, i.e., 0.203 eV. 

### 3.3. Photocatalytic Degradation of BPA by sP-MT Nanocomposite Membranes

#### 3.3.1. Influence of the Reaction Conditions and MWCNTs-TiO_2_ Content on BPA Removal

**Figure 7:**
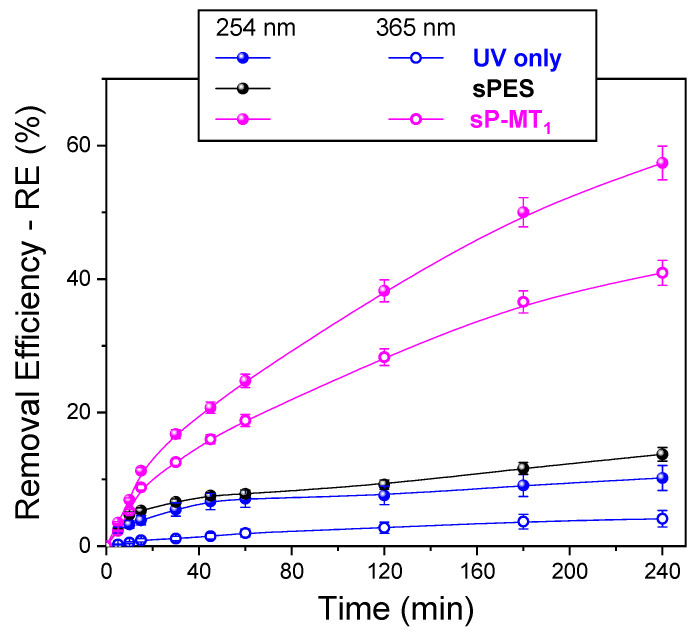
Preliminary investigation of BPA degradation for UV light, sPES, and sP-MMT_1_. Experimental conditions: V batch = 50 mL; [BPA] = 5 ppm; pH = 5.5.

The preliminary investigation of BPA degradation encompassed a range of experimental conditions to assess the combined impact of the UV light wavelength and the incorporation of the hybrid nano-catalyst on degradation efficiency. In this regard, Figure 7 offers a comparative analysis of the removal efficiency by UV rays only (two different wavelengths were tested, i.e., 254 and 365 nm) with that of sPES and one representative nanocomposite membrane, i.e., sP-MT_1_. In all the batch tests, the experimental parameters were maintained as follows: batch volume = 50 mL, pH = 5.5, and initial concentration of BPA = 5 mg/L. Both the UV light and pristine sPES membrane exhibit inadequate degradation efficiency, with levels below 15% for BPA degradation after 240 min, regardless of the wavelength used. Nevertheless, the introduction of a minimal amount of MWCNTs-TiO_2_ hybrid nanomaterial in the sPES matrix (i.e., 1 wt%) yields a considerable enhancement in removal efficiency. Indeed, BPA degradation escalated from nearly 14% for pristine sPES to an impressive 57% for sP-MT_1_ under UV light at 254 nm. This unequivocally underscores the exceptional photocatalytic potential of the MWCNTs-TiO_2_ nanohybrid. Xu and coworkers demonstrated that carbonaceous materials decorated with TiO_2_ combine three crucial features: (i) an enhanced visible light harvesting, (ii) more efficient charge separation, and (iii) improved adsorption ability for organics [97]. The synergistic effect of these features enables the MWCNTs-TiO_2_ material to yield remarkable photocatalytic ability and, thus, high BPA degradation. As expected, degradation efficiency declines using UV light wavelengths at 365 (approximatively 41% of BPA degradation). Consequently, to evaluate the effect of the filler content on the BPA removal efficiency, the samples were exposed to UV light at 254 nm.

**Figure 8:**
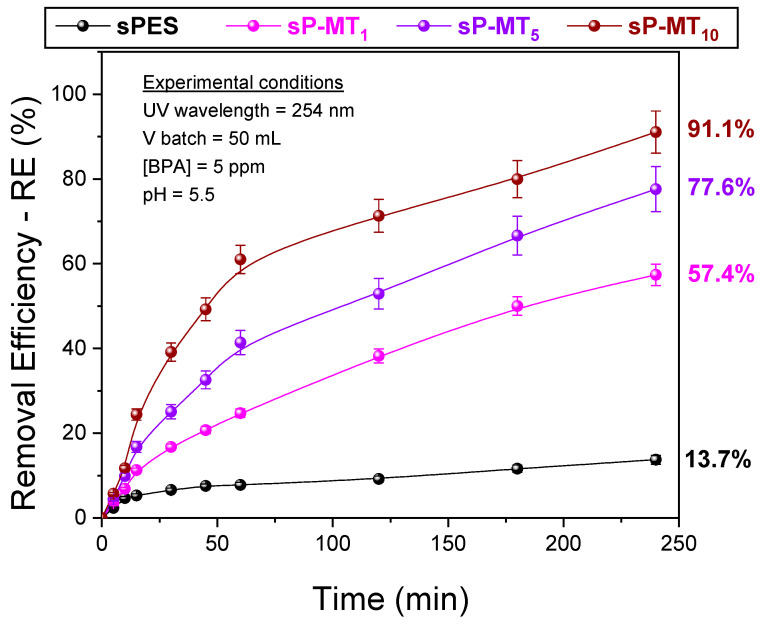
Removal efficiency of BPA as a function of time by sPES-based membranes.

As mentioned above, nanocomposite membranes at a higher filler content, i.e., 5 and 10 wt%, were also prepared, and their degradation efficiency was evaluated. Batch tests results with an initial BPA concentration of 5 mg/L (illustrated in Figure 8) show a degradation of BPA in an aqueous solution of 57.4%, 77.6%, and 91.1% after 240 minutes of UV treatment for nanocomposite membranes with 3, 5, and 10 wt% of MWCNT-TiO_2_ content, respectively. Moreover, the degradation kinetics also increase considerably with the dose of catalyst into the sPES. Indeed, the photodegradation became more efficient and rapid by increasing the concentration of the photocatalyst dose, going from about 30% for nanocomposite membranes with 1 wt% of nanofiller to 70% for sP-MT_10_ after only 120 min of treatment, thus underlining the substantial influence of enhanced active sites. Notably, it is noteworthy that even under UV irradiation at 365 nm, sP-MT_10_ achieves substantial BPA degradation of 82% (refer to Figure S2). Last but not least, sP-MT_10_ stands in close competition with the current state of the art, where a maximum BPA removal of 95% was reported after 240 minutes of treatment [98]. However, our nanocomposite membrane has clear competitive advantages, including the low TiO_2_ content, ease of recovery, and potential reusability. With the intention of further advancing scientific progress in this field and to offer enhanced solutions, additional investigations into both the effect of BPA concentrations and the reusability of the sP-MT10 nanocomposite membrane were undertaken.

#### 3.3.2. Effect of the Initial Concentration of BPA

**Figure 9:**
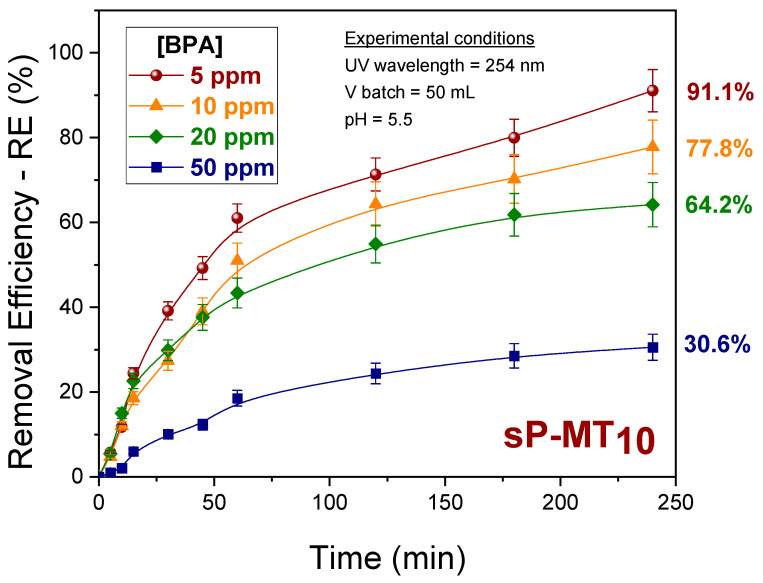
Removal efficiency of BPA vs. time by sP_MT_10_ nanohybrid membrane under different initial concentrations of BPA in water batches, i.e., from 50 to 5 ppm.

Exploring the impact of the initial BPA concentration on its degradation under UV light also constituted a focal point of this investigation. A series of experiments were carried out at pH = 5.5 using the sP-MT_10_ nanohybrid by subjecting it to 240 min of UV light exposure at a wavelength of 254 nm while varying the initial concentration of BPA from 5 to 50 ppm. The results are illustrated in Figure 9. The outcomes, depicted in Figure 9, underscore a clear dependence of both the extent and rate of BPA decomposition on its initial concentration. Higher BPA concentrations yielded reduced photocatalytic degradation, exemplified by 91.1% degradation for an initial concentration of 5 mg/L, which contrasts with a degradation efficiency of merely 30.6% for a concentration of 50 mg/L. These findings align well with observations made by Garg et al. [99], which indicated significant photolysis of BPA during medium-duration (2–4 h) irradiation. It is plausible that elevated BPA concentrations in the aqueous solution lead to adsorption on the membrane surface, thereby occupying active sites on the TiO_2_ catalyst. This phenomenon reduces the generation of hydroxyl radicals and affects the catalytic activity of the photocatalyst. Furthermore, higher BPA concentrations contribute to increased turbidity, consequently reducing the ability to effectively transmit UV light.

#### 3.3.3. Regeneration and Reuse of the Nanocomposite Membrane

**Figure 10:**
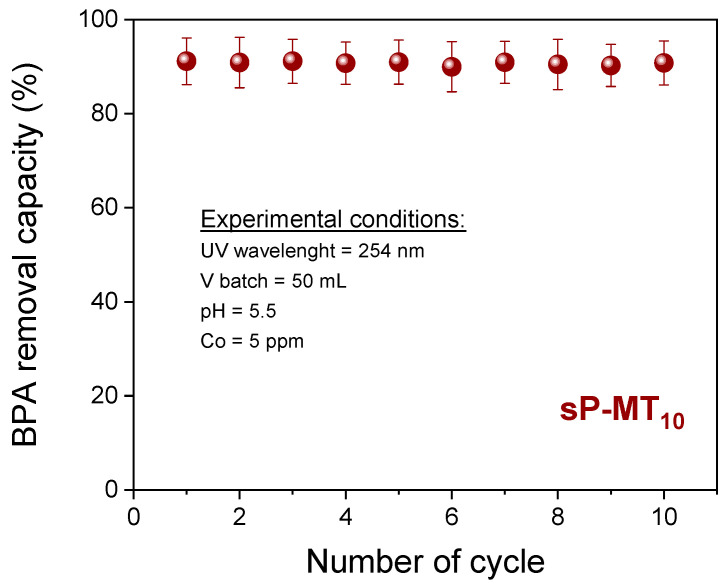
Bisphenol A removal efficiency, RE%, during regeneration and reuse experiments.

Considering the significant advantages associated with regeneration and reuse, such as reduced operational costs and minimized environmental impact, regeneration studies play a pivotal role in gauging the feasibility of large-scale application for this treatment methodology. For this purpose, sP-MT_10_ was subjected to multiple adsorption/desorption cycles, and its BPA removal efficiency was systematically evaluated. In each cycle, the membrane was removed from the water batch and subjected to a regeneration process involving 20 mL of ethanol (gradient grade for liquid chromatography, LiChrosolv^®^, Sigma-Aldrich) for a period of minutes under orbital stirring at 100 rpm and room temperature. Subsequent washing with distilled water was performed to eliminate any potential ethanol residues on the surface. This treatment allowed for cleaning the active sites of the catalyst, which were possibly covered by impurities generated in the degradation step, and making them available again for the UV degradation processes of BPA. After this simple, fast, and inexpensive treatment, the nanocomposite membrane was once again employed for BPA degradation under identical experimental conditions (initial concentration of BPA of 5 mg/L, pH = 5.5, and UV light at a wavelength of 254 nm). The results shown in Figure 10 unequivocally affirm the exceptional reusability of the nanocomposite membrane, with minimal reduction in its degradation capacity. Upon meticulous analysis, the decrease in degradation performance is negligible—merely 2%—after undergoing 10 consecutive use/regeneration cycles (decreasing from 91.1% to 89.8%). This underscores the remarkable stability and reusability of sP-MT_10_, particularly in contrast to the substantial performance drop of up to 50% reported in most literature materials after only five cycles [98].

## 4. Conclusions

A MWCNTs-TiO_2_ hybrid material was successfully synthesized via a facile hydrothermal method and tested as a nanofiller in a polymeric matrix of sulfonated polyether sulfone (sPES), resulting in the creation of novel nanocomposite membranes. These membranes hold significant promise as an efficient and scalable method for the degradation of bisphenol A (BPA) under visible light. Comprehensive characterization using XRD, Raman, and FTIR techniques has unequivocally established the covalent grafting of TiO_2_ nanoparticles onto the MWCNTs surface, thereby confirming the distinctive architecture of the MWCNTs-TiO_2_ hybrid material, with a TiO_2_ content of 47.6% estimated by TGA analysis. The incorporation of the hybrid material yields substantial enhancements in both the mechanical and hydrophilic properties of the sPES, translating to improved water mobility coupled with exceptional mechanical resistance. Intriguingly, the photocatalytic prowess of the pristine sPES is marginal, but it becomes increasingly substantial with higher filler loading, with sP-MT_10_ being able to achieve a BPA degradation of 91% and 82% after 240 min under UV irradiation at 254 nm and 365 nm, respectively. Furthermore, the results of regeneration tests underscore the chemical stability and remarkable reusability of sP-MT_10_. The sorbent can be efficiently regenerated through a swift washing process, showcasing no perceptible decline in BPA removal efficiency even after 10 cycles. Collectively, these findings converge to affirm the tremendous potential of this nanocomposite membrane for practical applications, offering a scalable, efficient, and stable solution for the photodegradation of BPA and/or other endocrine-disrupting chemicals.

## Data Availability

No new data were created.

## Supplementary Materials

The following supporting information can be downloaded at: https://www.mdpi.com/article/10.3390/nano13162325/s1, Figure S1. Cross-sectional SEM images of the sPES-based membranes at different filler loadings; Figure S2. BPA removal efficiency vs. time for sP_MT_10_ at two different UV wavelengths, i.e., 254 and 365 nm.
