# Supplementary material for: MWCNTs Decorated with TiO2 as Highly Performing Filler in the Preparation of Nanocomposite Membranes for Scalable Photocatalytic Degradation of Bisphenol A in Water
Source: Nanomaterials (Basel). 2023 Aug 13;13(16):2325. doi: 10.3390/nano13162325 (PMC10458988; doi:10.3390/nano13162325)
Supplement: Supplementary file 1 [file nanomaterials-13-02325-s001.zip › nanomaterials-2558415-supplementary.pdf]

nanomaterials

Supplementary Materials

MWCNTs decorated with TiO2 as highly-performing filler in the preparation of nanocomposite membranes for scalable photocatalytic degradation of Bisphenol-A in water

Antonio Tursi [1], Amerigo Beneduci [1,2], Isabella Nicotera [3,4], Cataldo Simari [3,4*]

Department of Chemistry and Chemical Technologies, University of Calabria, Via P. Bucci, Cubo 15D, 87036 Arcavacata di Rende, CS, Italy; antonio.tursi@unical.it (A.T.); amerigo.beneduci@unical.it (A.B.); isabella.nicotera@unical.it (I.N.)

[2] SIRiA S.r.l.-Servizi Integrati e Ricerche per l'Ambiente, c/o Department of Chemistry and Chemical Technologies, Spin-Off of the University of Calabria, Via P. Bucci, Cubo 15D, 87036 Arcavacata di Rende, CS, Italy

[3] National Reference Centre for Electrochemical Energy Storage (GISEL)—INSTM, Via G. Giusti 9, 50121 Firenze, Italy

* Correspondence: cataldo.simari@unical.it; Tel.: +39-0984-493385; Fax: +39-0984-492044

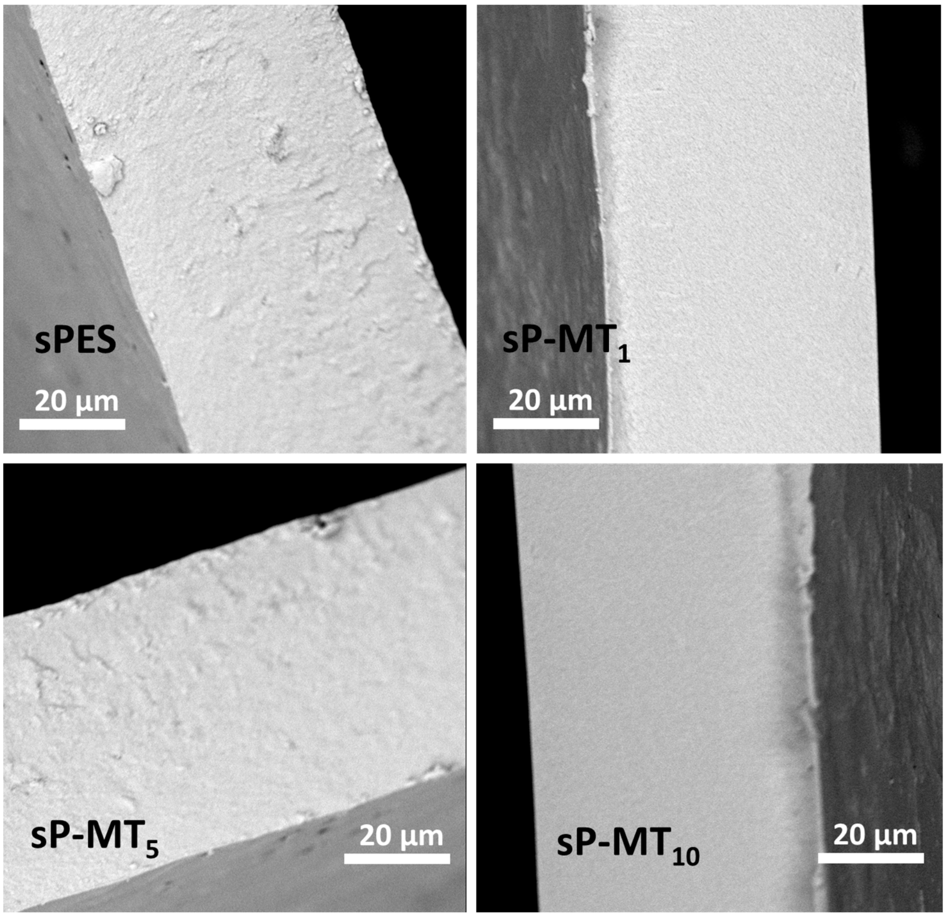

Figure S1. Cross-sectional SEM images of the sPES-based membranes at different filler loadings.

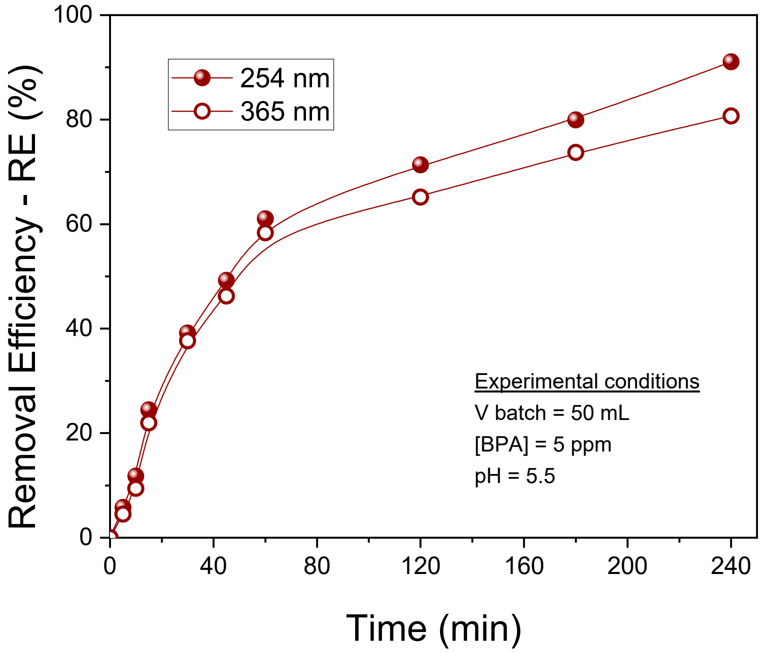

Figure S2. BPA Removal Efficiency vs. time for sP_MT10 at two different UV wavelength, i.e., 254 and 365 nm.
